# A resilience perspective on healthcare personnels’ experiences of managing the COVID-19 pandemic: a qualitative study in Norwegian nursing homes and home care services

**DOI:** 10.1186/s12913-023-10187-2

**Published:** 2023-10-28

**Authors:** Malin Knutsen Glette, David W. Bates, Patricia C. Dykes, Siri Wiig, Tone Kringeland

**Affiliations:** 1 SHARE – Center for Resilience in Healthcare, Faculty of Health Sciences, University of Stavanger, Stavanger, Norway; 2 Department of Health and Caring Sciences, Western Norway University of Applied Sciences, Haugesund, Norway; 3 Division of General Internal Medicine and Primary Care, Department of Medicine, Brigham & Women’s Hospital, Harvard Medical School, Boston, MA USA

**Keywords:** Primary healthcare personnel, Municipalities, Leadership, Quality of care, Allocation of resources, Elderly, Chronically ill, Personal protective equipment, Resilience, Adaptive capacity

## Abstract

**Background:**

The COVID-19 pandemic led to new and unfamiliar changes in healthcare services globally. Most COVID-19 patients were cared for in primary healthcare services, demanding major adjustments and adaptations in care delivery. Research addressing how rural primary healthcare services coped during the COVID-19 pandemic, and the possible learning potential originating from the pandemic is limited. The aim of this study was to assess how primary healthcare personnel (PHCP) working in rural areas experienced the work situation during the COVID-19 outbreak, and how adaptations to changes induced by the pandemic were handled in nursing homes and home care services.

**Method:**

This study was conducted as an explorative qualitative study. Four municipalities with affiliated nursing homes and homecare services were included in the study. We conducted focus group interviews with primary healthcare personnel working in rural nursing homes and homecare services in western Norway. The included PHCP were 16 nurses, 7 assistant nurses and 2 assistants. Interviews were audio recorded, transcribed and analyzed using thematic analysis.

**Results:**

The analysis resulted in three main themes and 16 subthemes describing PHCP experience of the work situation during the COVID-19 pandemic, and how they adapted to the changes and challenges induced by the pandemic. The main themes were: “PHCP demonstrated high adaptive capacity while being put to the test”, “Adapting to organizational measures, with varying degree of success” and “Safeguarding the patient’s safety and quality of care, but at certain costs”.

**Conclusion:**

This study demonstrated PHCPs major adaptive capacity in response to the challenges and changes induced by the covid-19 pandemic, while working under varying organizational conditions. Many adaptations where long-term solutions improving healthcare delivery, others where short-term solutions forced by inadequate management, governance, or a lack of leadership. Overall, the findings demonstrated the need for all parts of the system to engage in building resilient healthcare services. More research investigating this learning potential, particularly in primary healthcare services, is needed.

## Background

Immediately after the World Health Organization (WHO) announced that the Coronavirus disease (COVID-19) was a pandemic on March 12, 2020, healthcare services globally faced new and unfamiliar challenges [1, 2]. In addition to a major increase in patients needing acute care in the specialist healthcare services [3], primary healthcare services were forced to alter infection control and to prepare for infection outbreaks in nursing homes and among patients receiving home care services [4, 5].

Although the primary healthcare services are considered to represent the backbone of the healthcare service globally in almost all nations, it has often been “the poor stepchild” in terms of allocation of resources, competence, availability, and attention [6–8]. This was also seen in the beginning of the pandemic, as both infection control measures and distribution of personal protective equipment (PPE) were mainly focused on hospitals’ needs [7, 9]. However, only a small proportion of COVID-19 patients were hospitalized. Primary healthcare services managed the bulk of these patients, in addition to their important role in caring for, and protecting elderly and chronically ill patients from infection [8, 10]. Primary healthcare personnel had to make major adjustments to handle the ongoing pandemic, often despite deficient guidelines [5] and inadequate personnel [11].

Health personnel’s ability to adjust and adapt their work to expected or unexpected changes and maintain adequate performance is main pillars of resilience in healthcare (RiH) [12]. Taking a resilience perspective, health personnel need to make continuous adaptations to everyday changes or variations in the healthcare system [13]. While COVID represented an extreme example, these adaptations are critical. In this instance it included management of both performance variability, and management of complexity in terms of unexpected situations and variability in demands. This ability is, according to RiH, crucial to ensure adequate performance.

Although there is a growing body of research on how the primary healthcare services coped during COVID-19 [14–19], many issues remain unaddressed. Research has, for example, mostly focused on healthcare services situated in urban areas, leaving rural primary healthcare services under-investigated. There is also a specific need for evaluation in the Norwegian context, and for research aiming to learn from the COVID-19 pandemic [20].

### Aim and research question

In this study, we aimed to assess how primary care health personnel (PHCP) working in rural areas experienced the work situation during the COVID-19 outbreak. More specifically, the study aimed to explore how adaptations to changes induced by the COVID-19 pandemic were handled in nursing homes and home care services from the perspectives of PHCP. The study was guided by the following research question: *How did health personnel in rural primary care contextual settings experience the work situations during the COVID-19 outbreak and how did they adapt to the rapid onset changes induced by the COVID-19 outbreak?*

## Methods

### Design

This study was an explorative qualitative study. The qualitative design allowed for exploration of how changes induced by the COVID-19 pandemic were handled in the rural Norwegian primary healthcare services by obtaining an understanding of the meaning health personnel ascribed to the situation they found themselves in during the pandemic [21, 22].

### Setting

In Norway, municipalities are responsible for providing primary care to its inhabitants (e.g., nursing homes, homecare, care homes). A Norwegian municipality is a geographic delimited area that constitutes a separate political and administrative unit. Norway is divided into 356 municipalities with population rates ranging from 188 to 699,827 inhabitants [23]. The three smallest municipality types (which were the focus of this study) are characterized as *small-town municipalities, small center municipalities* and *least central municipalities.* They have an average population span from 1940 to 9601 inhabitants (Table 1.). The Norwegian municipalities are not fully autonomous but exercise autonomy within limits set by the parliament, this includes the operation of primary healthcare services. The municipalities and their affiliated primary healthcare services are organized and run differently, due to demographic, social and geographic conditions, which also causes differences in operating costs [24]. However, all municipalities are obligated to provide certain services (e.g., primary healthcare services) within a minimum standard [24].

**Table 1:** Definition of the three smallest municipality types in Norway

Definition	Number of municipalities in Norway	Average inhabitants	Highest number of inhabitants within the definition	Lowest number of inhabitants within the definition
Small town municipalities	96	9601	42,243	682
Small center municipalities	113	4342	13,180	844
Least central municipalities	125	1940	6346	208

### Recruitment

Small town, - small center, - and less central municipalities (Table 1) across the Norwegian west coast were invited to participate in the study. Health- and care managers of 11 municipalities were contacted via e-mail and invited to participate in the study. Four of these agreed to participate in the study and provided contact information to managers of nursing homes and homecare services within their municipality. The nursing home and homecare services managers were contacted by MKG and recruited to participate in the study. The same managers organized recruitment of health personnel within their services through distributing information letters and invitation to participate to their employees. The leaders also organized interview time and place in cooperation with MKG.

### Participants

Four municipalities and their nursing homes and homecare services were included in the study (Table 2). Half of the included municipalities had combined nursing homes and homecare services (the same health personnel covered both roles) due to their small size. The other half had one or several nursing homes and one homecare services covering the whole municipality.

The included PHCP were 16 nurses, 7 assistant nurses and 2 assistants with differing experience and (continued) education, all of whom worked during the COVID-19 pandemic (Table 2).

**Table 2:** Overview of participants

Municipality	Healthcare service	Health personnel	Size definition	Participants per municipality
Municipality A	Homecare services	4 Nurses	Small town municipality	7
	Nursing home	3 Nurses		
Municipality B	Homecare service & nursing home	1 Nurse 2 Assistant nurses 1 Assistant	Small center municipality	4
Municipality C	Homecare services	3 Nurses 2 Assistant nurses	Small center municipality	11
	Nursing home	3 Nurses 3 Assistant nurses		
Municipality D	Homecare service & nursing home	2 Nurses 1 Assistant	Less central municipality	3
Total number of participants				25

### Data collection

Data collection was conducted from September 2021 to November 2022. All interviews were conducted face to face with necessary infection precautions and measures. PHCP were interviewed in focus groups of 3–6 participants using a semi-structured interview guide based on previous research on health personnel and COVID-19 and RiH theory. The interviews were conducted by two researchers (MKG: moderator and TK: note taker). Both researchers had healthcare background in primary and secondary healthcare, and experience with qualitative interviewing (focus groups and individual interviewing). The interviews lasted for approximately 60 min, were audio recorded and transcribed verbatim.

### Data analysis

The data were analyzed using Braun and Clarkes thematic analysis [25], and was conducted by MKG. The transcribed data were read actively, and in several rounds to search for meanings or patterns. Text with meaning where then moved into a Word table and labeled with initial codes. When all text had been coded, 85 individual codes remained. The codes were read through carefully, and some were merged, separated into new codes or removed due to lack of relevance to the research question [25]. This resulted in 71 codes. In the next step, codes were analyzed to identify potential overreaching themes or subthemes. A new Word table was developed to help sort and find relationships between the different codes. When initial themes and subthemes were identified, a review was conducted to see if any themes needed to be “combined, refined and separated or discarded” [25]. (Table 3). Themes and subthemes were reviewed and approved by the author team.

**Table 3:** Overview of the analysis

Theme	Subtheme	Codes
**T1**: **PHCP demonstrated high adaptive capacity while being put to the test**	Insecurity, discomfort and being alone	Discomfort using PPE
		Feeling lonely
		Going to work, or staying home?
		Rewarded for the effort?
	Positive experiences	Being in control
		Feeling of work pride
		Experiences of own safety being safeguarded
		Support from colleagues and the local environment
		To have learned a lot
		Feeling of increased trust
	Fear of getting infected or infecting others	Fear of getting infected
		Fear of infecting others
		The relationship to their families and the world
	Experiencing infections firsthand	Getting infected at work
		Patients getting infected
	Feeling of Panic towards the unknown	Feeling of panic
		The unknown
		Lack of knowledge and training
	Health personnel are demonstrating multiple adaptive behaviors	Provisional solutions
		Prepared for every scenario
		Unpredictability
		Trust in self
		Make the best out of the situation
		Going the extra mile
		Creative and solution orientated
		Discretion
		Seeking information
	Handling new technology and/or tools	New tools
		New Technology
**T2: Adapting to organizational measures, with varying degrees of success**	Measures put in place to handle the pandemic was perceived as varying	Measures to avoid infections
		Measures perceived as useful
		Lack of, or confusing measures to avoid infections
		What to do better next time
		Measures to keep
		Measures perceived as bad
	Unfit buildings	Buildings not designed for infection control
	Issues regarding the staffing situation	Increase in work tasks and responsibilities
		Quitting and sick leave
		Fear of being moved to another ward
	Solutions for covering the staffing situation	Covering shifts
		Making staffing plans
		The use of other workers
		Remove “unnecessary “tasks
	Differences between neighboring municipalities	Better solutions in other facilities
		Lack of communication between the primary healthcare services
		Different rules in different municipalities
		Having enough PPE
		Not having enough PPE
	Difference in information perceptions in wards	Good information flow
		Challenging information flow
		Information overload
**T3: Safeguarding the patient’s safety and quality of care, but at certain costs**	Difficulties in obtaining compliance	Technology and elderly
		Lack of compliance among patients and family
	The patients were safe, but got their quality of life reduced	Effects of PPE on patients
		Patient’s quality of life got reduces
		Impersonal care
	Nurses worked targeted to maintain safety and quality	Measures to improve quality of life
		Caring for the patient’s safety
		Ensuring healthcare quality
		Taking care of the dead and their family

## Results

Three main themes describing how health personnel in primary healthcare experience the work situations during the COVID-19 outbreak and how they adapted to the rapid onset changes induced by the pandemic, were identified in the focus group interviews.

The findings showed that despite the uncertainty and unfamiliar challenges characterizing the COVID-19 pandemic, PHCP saw it as their duty to do what was needed of them, to keep the healthcare service running, including safeguarding healthcare quality and patient safety. The main challenges they encountered were reduced staffing, increased work tasks, facilities not built for infection control and an information overload. PHCP oversaw these challenges by, working extra hours, taking on extra work tasks, engaging in new roles, rearranging wards to ease infection control, developing tools to ease patients care, and find novel solutions to handle infection control in the facilities they had available.

### PHCP demonstrated high adaptive capacity whilst being put to the test

At the beginning of the pandemic, PHCP described being terrified of the new and unknown virus. They described how they distanced themselves from their spouses, children, and grandchildren, and worried about infecting their families or getting infected *by* their families, bringing COVID-19 into their workplaces. One nurse said that *she feared she would inflict an illness on someone from which they could potentially die.* Despite this fear, the PHCP embraced their responsibilities and went to work:

*I remember thinking that I had to go to work… many people could stay safe at home, but we couldn’t, we had to go to work. And I remember thinking, it is what it is, I must go to work, if I get it [COVID-19], if I die, then I’ll just have to be grateful for the life I’ve had so far…*.

(Nursing home, Municipality A)

As the world became more familiar with the virus and PPE, effective infection control routines and the vaccine came into place, health personnel described mainly feeling safe at work during the COVID-19 pandemic, although they felt more vulnerable to infection than others.

Although PPE was the main infection control measure providing security to the PHCPs, it was described as challenging to work with. They encountered problems like unbearable heat, foggy visors, and constant sweating. Despite wearing PPE, several of the PHCPs got infected with COVID-19 while at work. In one nursing home, almost every employee got infected, and experienced symptoms. Some of them were still struggling with the after-effects.

Due to the high infection rates and/or high amounts of quarantined PHCPs, the workload was, in several of the facilities, reported to be high. Some were forced to work 11-hour shifts and take on extra nigh-shifts with limited rest between them. They were required to take on extra workload to make up for the lack of personnel. Some were even called back from vacations or had planned vacations postponed. That said, some PHCP reported having almost no changes in their work schedules, demonstrating great variation between the included primary healthcare services.

Although fear, work schedules and PPE took a toll on the PHCPs, many also experienced positives from going through these challenges. Not only did they report that the pandemic had been a great learning experience (e.g., in infection control measures, PPE use, and managing critically ill patients) they also experienced an increased trust in their professional assessment by, for example attending physicians. Moreover, in addition to receiving support from the local community, many PHCPs reported that they experienced a good working environment where people supported, cared for, and helped each other as best they could:

*I have to say that we became wielded together handling something like this… we tried to make each other good, helped each other with things, helped each other getting dressed… it was really nice to see how we could be a team, and have fun together although going through something this tough.*

(Nursing home, Municipality C)

There were, however, also reports of loneliness, where the lack of joint lunch breaks, personnel meetings and generally being separated from colleagues – particularly in the homecare service, left PHCPs feeling alone. They missed being able to discuss things and solve professional problems with colleagues, and most of all they missed social interactions. There were also reports of PHCPs who had a hard time dealing with the high work pressure and made the decision to leave their positions during the COVID-19 period.

That said, PHCPs in all included primary healthcare services demonstrated how they adapted to the long-and short-term challenges they encountered in everyday work, despite the unpredictability (e.g., changing guidelines and routines) characterizing their workdays. They became creative in terms of findings ways to rearrange the wards to ease infection control, they designed their own tools (e.g., a patient record sheet for quick overview of patients) and became creative in how to make caring for infected patients easier and safer (e.g., use lockable cash boxes to able to keep the patients’ medications in their rooms). Homecare nurses changed their PPE in raw cellars, garages and on patients’ stairs and the nursing homes that did not experience any infected patients had provisional infection rooms and equipment ready, in case of infection outbreaks.

Despite the numerous pandemic related guidelines and rules PHCP had to deal with, they adapted guidelines to their professional discretion in many situations, particularly in situations where they cared for dying patients. They did for example make sure that patients had their family around them during their last hours (with necessary precautions and infection measures) despite visit restrictions, which did not occur in some other countries.

Moreover, PHCPs became much more accustomed to using electronic tools. Homecare nurses talked about an increase in use of medication dispensers for the patients’ homes. iPads and tablets were used for communication, both between patients and their families, and for having doctors’ visits and PHCP meetings. Most of the nursing homes had access to iPads or tablets before the pandemic but it pushed them to start to use them.

PHCPs from several of the included sites described that they stood up for the tasks ahead of them, and that they worked extra hours when needed. Terms like “going the extra mile”, “rolling up their sleeves” and “not backing out” were used.

### Adapting to organizational measures, with varying degree of success

Most PHCPs described that their task scope had increased. New tasks included disinfecting, arranging patient visits (with pre and post disinfections, registration, and assessment of risk of infection), care for extra patients, taking phone calls from worried next of kin and “guarding” patient rooms when patients suffering from dementia did not understand the isolation rules. In many of the primary healthcare services these extra work tasks were laid upon them without adding extra personnel. This felt chaotic and demanding for some.

It was quite early in the evening [the start of a night shift], and you had to be involved in the evening care of the patients [getting them ready for bed, a task dedicated for the evening shift]. And you might have to care for all the patients in the ward, because they [the evening shift], hadn’t had the time to do it. And then you work all night, without a break, because there’s not enough staff to take a break.

(Nursing home, Municipality A)

In the attempt to relieve work pressures, some adapted by dropping the least necessary tasks. For example, in homecare services they reduced supervision of patients that could do without it and tried to involve next of kin more in these tasks. Later, however, these measures were found to lead to a deterioration of some of the affected patients’ health.

A range of organizational adaptations to prevent spread of infection was described. Some primary healthcare services separated PHCPs into fixed groups and tried to keep these groups separated, although it was challenging to do so. They introduced pre-made meals for the patients, longer shifts (11 h), separated the break rooms, and gave courses in PPE use and infection control. Although primary healthcare services found that some of these measures worked, not all were appreciated or understood. Particularly in the beginning of the pandemic, measures were perceived to be unclear and confusing, such as when to use facemasks and which types were required. As the rules were unclear, some wore facemasks when caring for patients while others did not. There was also a limited access to PPE in the beginning of the pandemic and primary healthcare services’ conserved the PPE they had available to prepare for a potential COVID-19 outbreak. Moreover, there were reports of inadequate communication between cooperating healthcare services (e.g., home care service and ambulance personnel) in terms of infection risk among patients. In one case, ambulance personnel had been informed about a patient being an infection risk and showed up in full PPE when picking up the patient. The home care health personnel, however, had not been informed about this risk, and provided care to the patient without PPE. This caused insecurity, fear of contagion and a feeling of being undervalued.

Another reported problem was that there were different rules within different primary healthcare services, even within the same municipality. Moreover, there were differences in how distribution of information was managed in the different healthcare services. Although everyone agreed that there was a lot of information to comprehend, some participants were happy with the amount of information-, and how they got their information (often via e-mail or an information binder kept in the office) and managed to adapt accordingly. Others experienced an information overload which was difficult to oversee and struggled with interpreting the information as well as keeping track of the latest news.

There was also varying access to leaders during the pandemic period. Many described that they had leaders supporting their needs as best they could, who kept track of the information flow, and secured enough personnel. Others described that they had limited access to a leader.

*We lost a leader in this period… so we were… my ward was without a leader. We felt that everything was… complete chaos… Things were hanging midair…* (Municipality A).

### Safeguarding the patient’s safety and quality of care, but at certain costs

Overall, PHCPs in all included primary healthcare services perceived that the patient safety and quality of care was safeguarded during the COVID-19 pandemic. The aspect of safety and quality was said to be one of their highest priorities when adapting to challenges and changes induced by the pandemic.

The one main thing PHCPs believed to threaten quality of care, was the lack of psychosocial care the patients received. As a measure to avoid infection outbreaks in nursing homes, there were prolonged periods with firm visiting restrictions. This was perceived to severely affect patients’ quality of life. In addition, the PPE created a distance between the patient and the PHCP, limiting the interpersonal aspect of the care. Some nursing homes had the capacity to introduce measures to care for psychosocial aspects (and even employed personnel for this purpose), while others struggled to simply get their basic PHCP tasks done. In facilities where they had the resources to do so, PHCP took over the roles of next of kin and volunteers, coming up with creative measures to care for the patients’ psychosocial needs (e.g., arranging tea-parties, bingo, sing-alongs, making and showing videos from the patients’ families, work-out sessions and arranging “window visits”).

## Discussion

We evaluated the response of PCHPs to the COVID-19 pandemic in rural areas and found that it demanded an extreme level of adaption at a rapid clip. Moreover, we found that PHCPs had to make these adaptations under varying organizational conditions, and that the primary healthcare service often did not adapt as fast as PHCPs, for example, in terms of provision of rules and guidelines. The working environment, leadership, and health system organization profoundly affected PHCPs’ experience of working during the COVID-19 pandemic.

Previous research conducted on COVID-19 and primary healthcare services, supports our findings concerning the different challenges encountered by PHCPs—such as insecurity, the urge to self-isolate, staffing issues, changing guidelines, PPE shortage and information overload [11, 14, 26–28]. Staffing issues have been reported as a particular problem in smaller, more rural municipalities [29]. Although few studies in this context uses RiH theory as backdrop [2], several studies demonstrated PHCPs adaptive behavior in response to challenges and changes induced by the pandemic (e.g., increased use of electronic tools, workarounds, engaging in new responsibilities and roles) [27, 28, 30, 31]. Aspects identified in this study, which has been less illuminated in previous research included the role of professional discretion in the face of multitudes of new guidelines, challenges concerning varying guidelines in neighboring municipalities and healthcare services, and the challenging communication between collaborating healthcare services [32, 33] .

### How organizations affect PHCP adaptive capacity

Professional discretion is intricately linked to healthcare personnels adaptive capacity, which is one of the main pillars of RiH [12]. The span between professional discretion, adaptations and established rules and regulations, and how the different aspects are, or should be weighted in a resilient healthcare services remains underinvestigated [34]. RiH theory, usually describes resilient healthcare services as having the ability to adapt, improvise and being flexible - aspects highlighting the value of a decentralized authority allowing professional judgement and professional discretion, particularly when encountering high levels of variability, uncertainty and change [34]. This description of a resilient healthcare services nearly undermines rules and regulations, or at least, indicates a difficulty in reconciling them [34].

Although healthcare personnel are familiar with delivering healthcare in a context of constant social and political change [35], during the pandemic, the pace of healthcare delivery change was dramatic with major changes occurring often, along with rapid changes in rules and procedures. In many cases new regulations did not keep pace with the development of the pandemic, leaving a great responsibility on healthcare personnel and their professional discretion. In the current study, unclear regulation created frustration and insecurity among PHCPs, which in turn limited their adaptive capacity. This insecurity was particularly seen in the primary healthcare services which did not have a resolute leader. Later in the pandemic, however, when rules and guidelines became clearer, PHCP became more secure in making decisions based on professional discretion, and adapting guidelines to their professional assessment, for example “bending” the visiting rules for patients on their death beds.

On the other hand, previous studies have argued that too many rules and regulations and high external control restrain healthcare personnels’ adaptive capacity [36, 37]. According to Grote [38] there is a need to balance flexibility and stability to support RiH. The leaders play a key role in this context. On one side, healthcare personnel need the stability and security associated with established routines and rules, on the other side, they need their flexibility to adapt to the context they are working in, and to apply their professional discretion when providing healthcare to patients. In other words, a RiH promoting leadership needs to foster both cohesiveness and independence, thereby representing the dual, and often opposing challenge, of balancing flexibility and stability [38–40]. Moreover, leaders need to provide guidelines, structures and mechanisms that support resilience [34]. Providing such mechanisms and structures are, however, closely related to governance, particularly during crises [41, 42].

Governance “enables the financing, resource generation and service delivery functions to operate as intended and in coordination with the rest of the system to achieve maximum overall system performance, and by extension, resilience” [42]. Inadequate governance function results in confusing information flow, unclear guidelines, lack of equipment and staff, and lack of coordination between healthcare services [42]. This is supported by this and other studies [43, 44]. In the current study, there were examples of both well-functioning and less well-functioning governance. This variation was evident both across and within municipalities, however, the background for the variation remains unclear. One explanation may be related to the stringent division between municipal governance in Norway, where each municipality have the autonomy to organize and run their healthcare services at their own discretion, additionally, with different economic starting points. This will naturally lead to differences in healthcare organization in general, and COVID-19 management specifically, particularly if the cooperation and communication between the municipalities are limited. A recent literature review on resilience in healthcare during COVID-19 [2], found that the pandemic led to better and increased collaboration among healthcare services, and was found to be a contributing factor in maintaining resilient healthcare services, a finding supported by other literature [42, 45]. A report on the COVID-19 pandemic in Norwegian municipalities, however, found that intermunicipal collaboration was difficult due to differences in location (e.g., rural versus urban locations) and needs [43]. This indicates a need for more research investigating how the structure of primary healthcare services influence resilience in healthcare.

Rural healthcare services often face more challenges than urban areas (e.g., economic limitations, an elderly population, access to competence and large distances) [46, 47]. This can in turn limit their resilience in the face of healthcare crises, for example, in terms of a lacking supply chain, limited staffing during regular operation and the need to depend on more rural healthcare services for patients with critical conditions [48]. Although many of the aforementioned challenges were stated in the current study, there are large differences in how overreaching these challenges are in different municipalities, often regardless of size and rurality [47]. Overall, there are large variation in rural healthcare services both nationally and internationally, their sizes, location, their inhabitants, and their economy. This indicate inequality in terms of preconditions to handle crises, and may also be an explanation of the differences in COVID-19 management in Norwegian primary healthcare services [43]. Either way, it signifies the importance of a context disclosure when planning RiH enhancing work in primary healthcare services [49–51].

### Maintaining adaptive capacity over time

Despite both governance and leadership, healthcare personnel often adapt to their working conditions to provide quality care and safeguard patient safety, if they are provided with the flexibility to do so [14, 52]. This is reflected in this study through descriptions of adaptive capacity in all the included primary healthcare services, regardless of variation in both governance and leadership. As discussed earlier, PHCPs responsibility increased during the pandemic. They had to make more decisions on their own, in for example, how to organize wards, organize patient care and interpret guidelines and information from state level. These finding are supported by other studies [44]. Moreover, it can be argued that there was a higher need for PHCP adaptations as organization of hospitals got first priority in beginning of the pandemic [8, 53].

Anderson et al. [54] say that “adapting safely to pressure is what keeps the health system functioning.” However, there are different types of adaptations, varying from long term adaptations and innovations to unsustainable adaptations required in poorly functioning systems [52]. In this study we identified long-term adaptations such as development of new tools for quick patient overview and better organization of patient rooms to decrease infection risk. However, the varying conditions in the primary healthcare service during the pandemic also induced short-term adaptations, which of many, lead to unsustainable short-term solutions (e.g., taking on extra shifts to cover for the lack of staff and taking on tasks outside their work scope). Such adaptations are often caused by system unpreparedness, forcing healthcare personnel to find quick fix solutions to maintain healthcare quality [52]. At the pandemic peak, these measures were necessary to be able to keep the healthcare service running. However, over time, short-term adaptations are masking system failures and may lead to worn out PHCP, intention to leave, lower staffing and consequently decreased patient safety and care quality [37, 55].

The lengthiness of a crisis will often have an impact on healthcare resilience in terms of anticipating, coping, recovering and learning from the crisis [56]. Bardoel & Drago [57] proposes two types of resilience related to crisis’ over time: acceptance resilience (resource preserving) and strategic resilience (resource enhancing). *Acceptance resilience* involves continuing work as before, aiming at the same goals as before, but with greater costs of resources required to do so [57], for example PHCPs aiming to provide the same high-quality care to patients as before the pandemic, but with more tasks and less personnel. In contrast, *strategic resilience* invests in new environments, relationships and goals aiming to gain more resources when facing adversity, including planning new pathways appropriate for the new state of the system [57].

Although both strategies can be successful coping mechanisms, it is argued that strategic resilience is the most productive strategy when confronting a long-term crisis such as the COVID-19 pandemic [56, 57]. Förster et al. [56] claims that the healthcare services more often encounter short and intensive crisis’ (e.g., acute illness or injury) and therefore uses the “acceptance resilience” strategy more often when responding to unforeseen events. This is supported by Barasa et al. [41] who found that healthcare systems often focused on resilience in acute or catastrophic shocks to the system, and more seldom on chronic, everyday challenges. COVID-19 has forced health personnel and healthcare systems to focus more on long-term solutions. Long-term solutions may involve changes in health system operation and development of alternative routes to reach the desired goal (patient safety and quality of care) [18, 19, 41]. This stresses the importance of learning from the things that worked when handling the COVID-19 pandemic (e.g., fixed working groups, readymade meals for patients and dedicated personnel taking caring for patients sociopsychological needs), learning from the things that did not work (e.g., increase work scope without increasing staff, removing tasks involving direct patient care to reduce workload and rationing PPE) and take health personnel suggestions for improvement into consideration (e.g., keeping a PPE storage, build premises suited for infection control (also in primary care), more involvement of PHCP in decisions regarding reorganization, and better planning ahead of reorganization). According to RiH, learning from previous crises are critical for handling future ones more effectively [58]. Although predictions of future pandemics like COVID-19 vary, there seem to be an agreement among experts that the world is at high risk for pandemics in near future [59–61]. In this context, more research aiming to learn from the COVID-19 pandemic, both from what went well, and what did not, is needed for healthcare systems become more resilient and thus stay resilient during long-time burdens or crisis’ [62–64]. The COVID-19 pandemic offers a unique potential for this task.

This paper adds to the COVID-19 literature by demonstrating the major learning potential COVID-19 has in terms of building more resilient primary healthcare services. Considering the World Health Organizations’ aim of building more resilient primary healthcare services [62], more research untapping this potential is needed.

### Limitations

This study has certain limitations which should be considered. More municipalities from a wider area could have provided a broader perspective on PHCPs management of COVID-19 in rural healthcare services. There where however, relatively large variation in size, organization and rurality in the municipalities included, providing some variation in perspectives. Furthermore, due to COVID-19 restrictions, data were collected over time, implying that participants were at different stages of the COVID-19 development while being interviewed. This means that some PHCP had fresh experiences with the COVID-19 pandemic, while others had more retrospective experiences. What is more, there is a need to bear in mind the possible danger of diverting healthcare staff from patient care during the initial period of a crisis. However, the healthcare services set limits for this themselves by denying interview access until they had gained more control over the COVID-19 situation.

It is also important to consider that the PHCP were selected through their leaders. This strategy was implemented to enable the merging of interview time and work task at the respective wards yet, can generate issues such as undesirable pressure to participate or leaders selecting PHCP they think would provide “good” answers concerning the COVID-19 management. To limit this potential bias, the PHCP gave their final consent before the interview started and were given the opportunity to redraw from the study. The results indicate that recruited PHCP had varying experiences with the handling of the pandemic. Lastly, although the Norwegian perspective on PHCPs experiences of the COVID-19 pandemic in rural areas is lacking, adding additional countries would have strengthen the study.

## Conclusion

Primary healthcare personnel in this study demonstrated major adaptive capacity despite working under varying and trying organizational conditions. Many of the adaptions they described improved function, and delivered long-term adaptations, though some were work-arounds needed to cope with poor management, governance, or lack of leadership. We found that although PHCPs are a critical piece in the complex resilience puzzle, they cannot create a resilient primary healthcare service on their own. All parts of the system, including policymakers, regulators, leaders, healthcare personnel, and patients and their families need to contribute to this task. Moreover, rural healthcare services have varied context, this needs to be taken into consideration when building resilient primary healthcare services. Lastly, the study is an indicator of the learning potential inherent in a pandemic experience, which stands for one of the most extreme challenges a system can face and offers lessons regarding how to build future resilient primary healthcare services. More research investigating the learning potential of the pandemic is needed, as well as research exploring the importance of the interplay between all parts of the system in the development of resilient healthcare systems.

## Data Availability

Data are available from the corresponding author upon reasonable request.

## Abbreviations

PHCP: Primary healthcare personnel
RiH: Resilience in healthcare
PPE: Personal protective equipment
